# Immunotherapy for sepsis: From single-cell scenarios to clinical translation

**DOI:** 10.1016/j.gendis.2026.102220

**Published:** 2026-04-29

**Authors:** Zhengtao Zhang, Fang Xu

**Affiliations:** Department of Critical Care Medicine, The First Affiliated Hospital of Chongqing Medical University, Chongqing 400016, China

Sepsis has challenged medicine for centuries, and despite major advances since the germ theory era, it remains one of the most formidable problems in clinical immunology. Early successes in standardized sepsis management, based on bundled protocols of rapid antibiotic administration, aggressive fluid resuscitation, and organ support, yielded measurable reductions in mortality. However, in well-resourced settings, these improvements have plateaued, suggesting that a one-size-fits-all approach has reached its limits. This stagnation has prompted a fundamental re-evaluation of sepsis as a heterogeneous syndrome rather than a uniform condition. Recognizing and embracing this heterogeneity is now seen as essential for the next leap forward in sepsis therapy.

## Personalized immunotherapy in sepsis (ImmunoSep) test: Practice and limitations of precision immunological stratification in sepsis

In light of sepsis variability, precision immunotherapy has gained increasing attention by tailoring treatment to a patient's specific immune state (NCT03332225, NCT04990232, etc.). The foundation of this strategy lies in rapidly identifying an individual's immune phenotype and anticipating heterogeneous responses to immune modulation. A recent example is the ImmunoSep trial, a multicenter randomized study that attempted to match immunotherapies to patients' dominant immune profiles. Notably, this biomarker-guided approach led to a modest improvement in short-term organ dysfunction scores, suggesting that aligning therapy with immune status may confer clinical benefit.^1^ Yet, the trial also underscored the challenges of simplistic stratification. A substantial proportion of subjects did not cleanly fit the binary categories. Angus and others have argued that truly precision sepsis therapy must move beyond coarse immune grouping and instead select immunotherapeutic agents and dosing strategies based on multidimensional immune profiles rather than relying on fixed treatment protocols. Despite its limitations, ImmunoSep stands as an important milestone in the journey toward precision immunotherapy in sepsis, suggesting the potential for improved outcomes when treatments are guided by immune phenotype.^2^

## Single-cell multi-omics technology reshapes the understanding of immunological heterogeneity in sepsis: From binary models to dynamic multidimensional profiling

**Figure 1:**
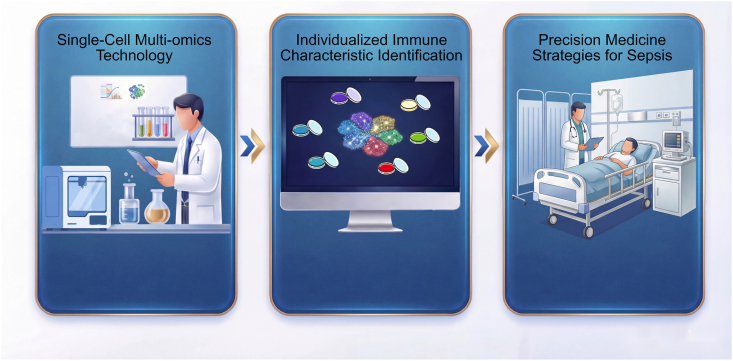
Single-cell multi-omics analysis of individual immune characteristics drives precise stratification and treatment of sepsis.

Recent studies have illustrated how single-cell analyses can uncover previously unappreciated immunological heterogeneity in sepsis^3^ (Fig. 1). Our single-cell comprehensive dataset revealed both shared and patient-specific immune signatures across different age groups and infection sites. One notable finding was an NR4A2^+^ central memory CD4^+^ T cell subset enriched in patients with certain infections (abdominal, pulmonary, and cutaneous soft tissue sepsis) that exhibited an exhausted phenotype. Functional experiments implicated this subset in sepsis pathogenesis, as conditional knockout of *Nr4a2* in CD4^+^ T cells improved survival in septic mice, whereas overexpression of this factor exacerbated inflammation and organ injury. Single-cell analyses from our group further highlighted context-dependent immune features. In adult patients with abdominal or pulmonary sepsis, our data showed an expansion of proinflammatory cytotoxic lymphocyte populations, including CD8^+^ T, NK, and NKT cells, which were characterized by elevated expression of CCL3, CCL4, and TNF-α. By contrast, pediatric patients with sepsis due to pneumonia showed a predominant expansion of proliferative CD14^+^ monocytes. Collectively, such single-cell insights provide a more nuanced framework for patient classification and suggest that each sepsis case may represent a distinct immunological subtype.^3^ Importantly, this granular understanding opens the door to identifying targetable cell populations or pathways that would have been overlooked by bulk analyses or by rigid binary models.

It should be noted that studies applying single-cell transcriptomics in sepsis remain at an early stage of development. Most are based on single-center cohorts and limited temporal sampling. To fully realize the potential of these technologies, future investigations will need to incorporate larger, longitudinal designs that track the evolution of immune states from disease onset through recovery or death. Expanding analyses to include additional immune compartments will provide a more comprehensive view of sepsis immunology. Furthermore, the integration of data from independent multicenter cohorts will be essential for establishing robust and generalizable predictive models of immune trajectories.

## Transcriptomic subtyping of sepsis: From a tri-subtype consensus framework to precision subtyping via multimodal integration

A recent study by Scicluna and colleagues integrated whole-blood transcriptomes from two large sepsis cohorts, namely, the Netherlands MARS study and the United Kingdom GAinS study, to derive three consensus transcriptomic subtypes of sepsis, designated CTS1, CTS2, and CTS3. This consensus framework reconciled previously disparate gene expression classifications by demonstrating substantial overlap and refining them into a reproducible three-subtype structure. Each subtype corresponds to a distinct immunopathological pattern. Collectively, this transcriptomic framework provides a structured approach for interpreting molecular heterogeneity in sepsis and offers a potential basis for patient stratification in clinical trials.^4^

Ongoing investigations are exploring how additional data modalities, including proteomic, metabolomic, and single-cell analyses, may refine or expand current classifications. Ultimately, the aim is to develop a clinically actionable taxonomy in which an individual patient's immune profile can be mapped to a defined endotype to inform therapeutic decision-making.

## From dualism to personalized therapeutic targets: A clinical translation framework for precision immunological stratification of sepsis

Encouragingly, the conceptual foundations for immune-based patient stratification are being established across both research and clinical domains.^1^^,^^3^ Sepsis involves a dynamic and concurrent interplay of immune processes that cannot be adequately described by a simple sequential or binary framework. In this context, the concept of individualized treatable traits has emerged as a potential organizing principle for precision immunotherapy.^5^ This approach envisions a cycle of stratification, intervention, and reassessment. Compared with static protocol-based care, this strategy acknowledges that immune status in sepsis is dynamic and context dependent. To translate this conceptual framework into practice, several advances will be required. Multimodal evidence from translational research should be systematically incorporated to refine the definition of treatable traits and to establish thresholds for intervention.^5^ Although this agenda is ambitious, it is increasingly feasible as technological and analytical capacities expand.

Looking ahead, the integration of single-cell analytics with systems immunology and precision medicine frameworks may facilitate a more structured and responsive approach to sepsis management. Rather than relying solely on empirical bundled therapies, future strategies may incorporate immune state-guided interventions informed by dynamic profiling. Continued refinement of immune stratification models has the potential to improve therapeutic alignment and, ultimately, clinical outcomes in sepsis.

## CRediT authorship contribution statement

**Zhengtao Zhang:** Writing – original draft, Resources. **Fang Xu:** Writing – review & editing, Resources, Project administration.

## Conflict of interests

The authors declare no conflict of interests.

## References

[1] Giamarellos-Bourboulis E.J., Kotsaki A., Kotsamidi I. (2026). Precision immunotherapy to improve sepsis outcomes: the ImmunoSep randomized clinical trial. JAMA.

[2] Angus D.C. (2026). Precision therapy for sepsis: the end of the beginning?. JAMA.

[3] Ye Q., Lai X., Liu Y. (2026). Single-cell multi-omic landscape reveals anatomical-specific immune features in adult and pediatric sepsis. Nat Immunol.

[4] Scicluna B.P., Cano-Gamez K., Burnham K.L. (2025). A consensus blood transcriptomic framework for sepsis. Nat Med.

[5] Giamarellos-Bourboulis E.J., Aschenbrenner A.C., Bauer M. (2024). The pathophysiology of sepsis and precision-medicine-based immunotherapy. Nat Immunol.

